# Supplementary material for: Patterns of Intron Gain and Loss in Fungi
Source: PLoS Biol. 2004 Nov 30;2(12):e422. doi: 10.1371/journal.pbio.0020422 (PMC532390; doi:10.1371/journal.pbio.0020422)
Supplement: Table S1 — Also available at http://genes.mit.edu/NielsenEtAl/. (4.3 MB ZIP). [file pbio.0020422.st001.zip › NielsenEtAl/html/621.html]

AN5616.1.NCU03347.1.MG03264.1.FG01560.1


```
 CLUSTAL W (1.82) Multiple Sequence Alignments - Introns Inserted


Sequence 1: NCU03347.1	423 aa
Sequence 2: MG03264.1	426 aa
Sequence 3: AN5616.1	418 aa
Sequence 4: FG01560.1	426 aa
Alignment Length: 429 aa
Number Identitical Residues: 250 aa
Alignment Score (without introns) 12676


MG03264.1 	MSTGSAK---FQPAARVAGRRQDVW2SIINEAATASPKQPIVNMGQGFF~GYNPPDFILN
NCU03347.1	---MSAK---LKPAARVAGNRQDVW2TIVNEAAAASPRQPIVNMGQGFF~GYNPPEFILN
FG01560.1 	MSNQTQK---LQPAGRVQGQKKDVW2SMINEAAASSPIQPIVNLGQGFF~GYNPPDFILN
AN5616.1  	MSATTARSDPFKPAARVAGQRQDVW2SIVNEAAAASPVQPIVNMGQGFF2GYNPPQFALD
          	 :  : ::..::**.** *.::*** :::****::** *****:***** *****:* *:

MG03264.1 	AAKQALDKVDCNQYSPTK~GRPRLKKAIADAYAPFWGRQLNPDTEVTITTGANEG1MLSA
NCU03347.1	AAKKALDRVECNQYSPTR~GRPRLRKALSKAYAPHWGRELDPETEITITTGANEG~MLSA
FG01560.1 	AAKEALDRVECNQYAPAK~GRPRLRQALADAYSPSWGRQLDPETEIIITTGANEG~MLSA
AN5616.1  	AAKAALDRVDCNQYSPTK0GRPRLKKALADAYSPFFGRQLNPDTEVTITTGANEG1MLSA
          	*** ***:*:****:*:: *****::*::.**:* :**:*:*:**: ******** ****

MG03264.1 	FMAFIEPGDEVIIFEPFFDQ2YISNIEMPGGKIVYVPLHPPTEGATKTLSAGAWNIDFDE
NCU03347.1	FMAFIEPGDEVIVFEPFFDQ~YISNIEMPGGKIVYVPMHPPKEGHIKTLSASEWTIDFDE
FG01560.1 	FMGFIEAGDEVIVFEPFFDQ2YISNIQMPGGKVVYVPLHPPKTGATKNSSAADWTIDFEE
AN5616.1  	FMGFIEQGDEVIIFEPFFDQ2YISNIEMPGGTIRYVPLQPPKDGATKTLPASEWSINFDE
          	**.*** *****:******* *****:****.: ***::**. *  *. .*. *.*:*:*

MG03264.1 	LERAITPKTKMI~VLNTPH~NPVGKVFTKDELQKIADLCLKNEIIILSDEVYDRLFYTPF
NCU03347.1	LERAVTPRTKML~VINTPH~NPVGKVFSRAELERIAELCVKNQIIILSDEVYDRLFYTPF
FG01560.1 	LEKAFTPRTKMI~VINTPH1NPVGKVFSKEELQGIADLAVKHQVIILSDEVYDKLYYVPF
AN5616.1  	LERTINPKTKMI0V-----~------FSRDELERIGDLAVKHNLIILSDEVYDRLYYVPF
          	**::..*:***: *            *:: **: *.:*.:*:::*********:*:*.**

MG03264.1 	TRIATLSPEVERITLTVGSAGKNFYATGWRVG1WLMGPAELIQHVSAAHTRICYSSVSPL
NCU03347.1	TRMATLSPEVEKITLTVGSAGKNFYATGWRVG~WLMGPAELIQYVAAAHTRICYSSVSPL
FG01560.1 	TRIANLSPEVEKLTLTVGSAGKNFYATGWRVG1WLIGPQELIQYVTAAHTRICFSTPAPF
AN5616.1  	TRIATMKPEYYERTLTVGSAGKAFYATGWRVG~YLIGPEHLIKYVAGAHTRICYSSVSPL
          	**:*.:.**  . ********* ********* :*:** .**::*:.******:*: :*:

MG03264.1 	QEACAVG1FEQAEKEGFWDECVRDMKRRMDLFNQVWHELGIP~FSEPEGGYFVMVNLSTV
NCU03347.1	QEACAIG~FEEADQQGFWTQCIEDMQNKMSRFNAIWDELGLP~YSIPEGGYFVMANLNKV
FG01560.1 	QEASAIG~FEQAGKNGFWDETIKEMKGKIDRLKEVFEELELP~VTYPEGGYFLLVNMAKV
AN5616.1  	QEAAAVA~FEEADKQGFWDQSREEMKRKMERFCEVFDELNIP0YSDPEGGYFVLANMASV
          	***.*:. **:* ::*** :  .:*: ::. :  ::.** :*  : ******::.*: .*

MG03264.1 	QMPENYPFPPHVASRPRDFKLAWFLIQELGVAAIPPTEFFTDANAHIVEDYLRFAVCKPD
NCU03347.1	EIPEGYPFPPHVAERPRDFKLAWFLIQELGVAAIPPSEFYTDVNAHIVEDWLRFAVCKPD
FG01560.1 	KLPEDYPFPPHVASRPRDFKLAWFLIQEIGVAAIPPTEFYTPVNAHLAEDYIRFAVCKND
AN5616.1  	KLPEGYPFPPHVASRPRDFKLCWFLIHEVGVAAIPPTEFYTDANAHIAEDYLRFAVCKED
          	::**.********.*******.****:*:*******:**:* .***:.**::****** *

MG03264.1 	DVLELAKERLRGLKKFIKQ
NCU03347.1	EVLEDAKERLRGLKKFIKQ
FG01560.1 	DILEQAKERLRGLKKYIPE
AN5616.1  	DVLETAKERLRGLKKYIVR
          	::** **********:* .
```
